# Prevalence and grade of scapular dyskinesis in patients with shoulder injuries: a cross-sectional study

**DOI:** 10.1016/j.jseint.2026.101643

**Published:** 2026-01-29

**Authors:** Ban Suk Sim, Young Kyun Kim

**Affiliations:** Graduate School of Sports Medicine, CHA University, Phocheon, Republic of Korea

**Keywords:** Scapular dyskinesis, Shoulder injury, Cross-sectional study, Epidemiology, Shoulder pain, Prevalence

## Abstract

**Background:**

Scapular dyskinesis (SD) is an impairment that can be associated with shoulder injuries; however, its prevalence and grade has not been thoroughly investigated. Therefore, this study aimed to investigate the prevalence and grade of SD in patients with shoulder injury.

**Methods:**

In total, 210 patients with shoulder injury participated in this study between May and July 2023. Shoulder injuries were diagnosed, and SD was assessed in injured and noninjured shoulders. The shoulder injury duration was also assessed.

**Results:**

In total, 205 patients (97.6%) had SD with injured shoulders, and 115 patients (54.7%) had Grade 3. Among the noninjured shoulders, 69% (n = 145) had SD and 6.7% (n = 14) had Grade 3. Patients with a duration of shoulder symptom >6 years had an incidence of Grade 3 of 100%.

**Conclusion:**

The prevalence of SD was significantly high in injured shoulders compared with noninjured shoulders. Moreover, the grade of SD in injured shoulders was severe compared with that in noninjured shoulders. SD may increase dysfunction in injured shoulders; therefore, it is recommended to assess and treat SD in patients with shoulder injury.

Scapular dyskinesis (SD) is defined as altered scapular motion and/or position.^19^ Although SD is not an injury, it has been related to shoulder injuries.^19^^,^^46^ Shoulder impingement, rotator cuff tears, superior labral injuries, acromioclavicular (AC) joint separation, multidirectional instability, scapular muscle detachment, clavicle fracture, snapping scapula, and neurological injuries are related to SD; however, the exact etiology remains unclear.^17^^,^^19^ Various sports related to SD have been previously studied, and the prevalence of SD in overhead athletes is twice as common (61%) as that in nonoverhead athletes (33%).^1^ Rotator cuff injuries, impingement syndrome, and frozen shoulder with scapular muscle activation and movement, or scapular static position, have been reported. However, studies on SD prevalence in nonathletic populations with shoulder injuries are lacking. Scapular kinematic alterations, also known as SD, can arise from both physiological factors, such as soft tissue tightness, muscle weakness, or pain-related inhibition, and anatomical factors, including AC joint injury, rotator cuff tear, or clavicle fracture.^16^ These alterations are commonly associated with increased upper trapezius activity, reduced serratus anterior activation, pectoralis minor tightness, and thoracic kyphosis,^19^^,^^24^ highlighting the multifactorial nature of scapular dysfunction. Shoulder impingement patients with Grade 3 have been reported to present with reduced scapular external rotation and increased upper trapezius muscle activity during shoulder flexion.^22^ Glenohumeral instability and impingement result in scapular malalignment.^46^ Rotator cuff pathology shows altered shoulder kinematics, and full-thickness rotator cuff tear is associated with greater scapular elevation.^27^ Adhesive capsulitis results in increased scapular lateral rotation.^7^ The inhibitory effect of pain on muscle activation might disrupt the normal activation of periscapular muscles^16^ and it might be related to SD. Evidence of SD with shoulder injuries has been reported; however, the prevalence and grade of SD with shoulder injuries have been poorly investigated.

Grade 3 is associated with an increased risk of shoulder injuries,^3^ and specific shoulder injuries may be negatively influenced by the presence of SD.^31^ Thus, SD should be evaluated and treated when shoulder injury is present because SD could impair shoulder healing.^23^^,^^31^ Therefore, this study aimed to investigate the prevalence and grade of SD in patients with shoulder injuries. The primary objective was to compare the prevalence of SD in injured shoulders and the grade of SD in noninjured shoulders. The secondary objective was to compare the grade of SD according to the chronicity of the shoulder injury.

## Materials and methods

### Ethics statements

This study was approved by the institutional review board of CHA University (1044308-202303-HR-080-02), and informed consent was obtained from all the patients.

### Study design and participants

**Figure 1:**
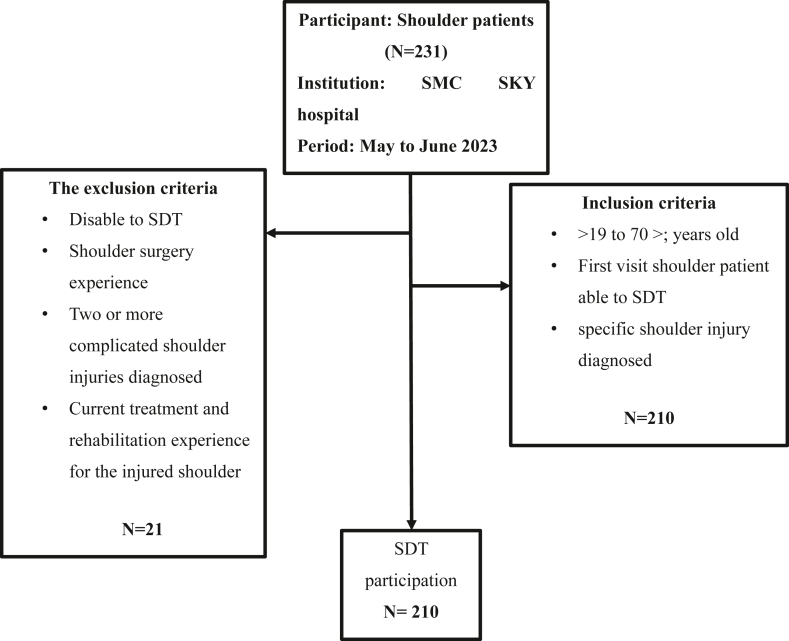
CONSORT flow diagram for SDT showing patient recruitment, exclusion, and participation in the SDT. A total of 231 patients were recruited; 21 were excluded, resulting in 210 patients analyzed. *CONSORT*, Consolidated Standards of Reporting Trials; *SDT*, scapular dyskinesis test; *SMC SKY,* sports medicine center SKY Hospital.

The total sample size was determined using G∗Power 3.1 (Heinrich Heine Universität, Düsseldorf, Germany), resulting in a calculated sample of 231 participants with effect size of 0.4 and power value of 0.92. We diagnosed (n = 210) patients with shoulder injuries who volunteered for this cross-sectional study at sports medicine center SKY Hospital (Seoul, Korea). A total of 231 patients were recruited from May to July in 2023; however, 21 patients were excluded because of their inability to perform the scapular dyskinesis test (SDT) (Fig. 1). The inclusion criteria were as follows: (1) age >19 to <70 years and (2) first-visit patients with shoulder injuries who were able to undergo a SDT with a specific shoulder injury diagnosed. The exclusion criteria were as follows: (1) inability to perform a SDT, (2) shoulder surgery experience, and (3) two or more shoulder injuries diagnosed at the same time, or current treatment and rehabilitation experience for the injured shoulder.

## Measures

### Diagnosis of shoulder injuries

Clinical examination, radiography, ultrasonography (US), and magnetic resonance imaging (MRI) were used to diagnose shoulder injury. To diagnose shoulder impingement syndrome and Neer and Hawkins impingement sign were assessed. Radiographic evidence essential for diagnosis includes subacromial space narrowing and concomitant abnormal acromial morphology, and MRI was used to assess inflammation in the subacromial bursa.^4^ Painful arc, the presence of a drop arm sign, infraspinatus test was performed in prone with the shoulder at 90° abduction and the forearm unsupported. External rotation strength was assessed by stabilizing the elbow and applying resistance at the distal forearm^44^ and US and MRI findings were used to diagnose rotator cuff pathology.^34^^,^^45^ Rotator cuff calcification was evaluated using standard shoulder radiographs. Anteroposterior views in internal and external rotation, along with an axillary lateral view, were obtained to determine the location of the calcific deposits within the tendon.^8^ MRI, history, range of motion (ROM), and active compression test were used to accurately diagnose superior labral anteroposterior lesion (SLAP).^18^ Painful passive external rotation, radiography findings, and stiffness indicated adhesive capsulitis, and then radiography and US were used to rule out other possible injuries.^2^^,^^10^ Asymmetry of the shoulder, active and passive ROM, apprehension, sulcus, and load and shift test results were used to diagnose recurrent dislocation.^6^ The period of the shoulder injury was obtained from the patient to record the chronicity. All shoulder diagnoses were made by a physician with >20 years of experience in orthopedics and sports medicine.

### Scapular dyskinesis test

The SDT was used to assess SD. The physiotherapist with 7 years of experience who performed the SDT was blinded to the shoulder diagnosis and the injured side. The patient was instructed to stand in a neutral position with the elbows straight. If patients weighing ≤68.1 kg were asked to hold 1.4-kg (3-lb) dumbbells in both hands, while those weighing >68.1 kg held 2.3-kg (5-lb) dumbbells during the SDT. The patient was asked to flex his/her shoulders to 180° for 3 seconds and then asked to return to a neutral position for 3 seconds, five times, with both elbows straight.^26^ The examiner palpated both scapulae with both hands during the SDT to grade SD. SD was graded using a three-point scale: Grade 1 (normal), no observable abnormality; Grade 2 (subtle), mild or questionable abnormality; and Grade 3 (obvious), clearly apparent abnormality.^11^^,^^26^ The reliability of the SDT in a previous study was moderate to substantial (k = 0.64),^11^ and the examiner of this study also showed substantial agreement (k = 0.72). The grade of SD was used to analyze the relationship with shoulder injuries, noninjury side, and chronicity of the shoulder symptom.

### Statistical analysis

Descriptive statistics were used to analyze the grade of SD according to shoulder injuries and periods. The chi-square test was used to compare the grade of SD between the injured and noninjured sides. The level of significance was set at *P* < .05. All statistical analyses were performed using R (version 4.3.1 for Windows; R Foundation for Statistical Computing, Vienna, Austria).

## Results

### Prevalence of scapular dyskinesis with shoulder injuries

Shoulder injuries were diagnosed in 210 patients, of whom 205 (97.6%) had SD with injured shoulders (Table I). Grade 3 was observed in 115 patients (54.7%), and G2 was observed in 90 patients (42.9%). Only five patients (2.4%) showed Grade 1 within the injured shoulders. Eighty-six (41%) patients were diagnosed with shoulder impingement syndrome, and of those, 83 patients (96.5%) showed SD. All patients with supraspinatus calcification (n = 30) and supraspinatus tendinitis (n = 26) had SD. Patients with supraspinatus partial tear (n = 25) showed a prevalence rate of 96% for SD. Twenty patients with SLAP showed a prevalence rate of 100% for SD. Adhesive capsulitis (n = 13), recurrent dislocation (n = 4), infraspinatus tendinitis (n = 3), and infraspinatus partial tear (n = 3) were diagnosed with high rates of SD (92.4% with adhesive capsulitis and 100% with recurrent dislocation, infraspinatus partial tear, and tendinitis).

**Table I:** The prevalence of scapular dyskinesis with shoulder injuries.

Shoulder injury	n (%)	Grade 1 (%)	Grade 2 (%)	Grade 3 (%)
Impingement syndrome	86 (41)	3 (3.5)	41 (47.7)	42 (48.8)
Supraspinatus calcification	30 (14.3)		12 (40)	18 (60)
Supraspinatus tendinitis	26 (12.4)		12 (46.2)	14 (53.8)
Supraspinatus partial tear	25 (11.9)	1 (4)	8 (32)	16 (64)
SLAP	20 (9.5)		7 (35)	13 (65)
Adhesive capsulitis	13 (6.2)	1 (7.6)	6 (46.2)	6 (46.2)
Recurrent dislocation	4 (1.9)		1 (25)	3 (75)
Infraspinatus partial tear	3 (1.4)		1 (33)	2 (67)
Infraspinatus tendinitis	3 (1.4)		2 (66.7)	1 (33.3)
Total	210	5 (2.4)	90 (42.9)	115 (54.7)

*SLAP*, superior labral anteroposterior lesion.

### Grade of scapular dyskinesis in injured shoulders

The grade of SD in injured shoulders was significantly higher than that in noninjured shoulders (*P* = .005) (Table II).

**Table II:** Comparison of the severity of scapular dyskinesis in patients with injured and noninjured shoulders.

Shoulder injury	N	Injured side	Noninjured side	Chi-square	*P* value
		Median	Median		
	210	3	2	72.141	.005∗

*SD*, scapular dyskinesis.

SD was graded as Grade 1 and Grade 2or Grade 3. Data are expressed as frequency (%). Chi-square test was used for comparison.

Grade: 1 = normal, 2 = subtle, 3 = obvious.

∗ Statistically significant.

### Prevalence of scapular dyskinesis in noninjured shoulders

The prevalence of SD in injured and noninjured shoulders was 97.6% and 69%, respectively (Table III). Grade 3 was observed in 54.7% of injured shoulders and 6.7% of noninjured shoulders. Grade 2 was seen in 42.9% of injured shoulders and 62.4% of noninjured shoulders. Grade 1 was observed in 2.4% of injured shoulders and 30.9% of noninjured shoulders.

**Table III:** Comparison of the severity of SD between injured and noninjured shoulders.

	Grade 1 (%)	Grade 2 (%)	Grade 3 (%)	Prevalence of SD (%)
Injury side	5 (2.4)	90 (42.9)	115 (54.7)	205 (97.6)
Noninjury	65 (30.9)	131 (62.4)	14 (6.7)	145 (69)

*SD*, scapular dyskinesis.

### Grade of scapular dyskinesis with the chronicity of shoulder symptom

Among patients presenting with a shoulder injury with a symptom duration of less than 6 months (n = 88), 2 (2.3%) exhibited Grade 1, 40 (45.5%) showed Grade 2, and 46 (52.2%) demonstrated Grade 3 (Table IV). Among patients with shoulder symptom (n = 42) during the 6 months to 1-year period, 1 (2.4%), 19 (45.2%), and 22 (52.4%) showed Grade 1, Grade 2, and Grade 3, respectively. Among patients with shoulder symptom during the 1-2-year period (n = 46), 1 (2.2%), 21 (45.6%), and 24 (52.2%) exhibited Grade 1, Grade 2, and Grade 3, respectively. Among the patients with shoulder symptom (n = 26) during the 2-5-year period, 1 (3.8%), 10 (38.5%), and 15 (57.7%) showed Grade 1, Grade 2, and Grade 3, respectively. During the 6-10-year period, all patients with shoulder symptom (3, 100%) showed Grade 3, and over the 10-year period, all patients with shoulder symptom (5, 100%) exhibited Grade 3. The average grades of SD were 2.48 at 0-6 months, 2.32 at 6 months to 1 year, 2.61 at 1-2 years, 2.25 at 2-5 years, 3 at 6-10 years, and 3 at >10 years.

**Table IV:** Comparison of chronicity of the symptom side with scapular dyskinesis over time.

Chronicity of the injured side						
	0-6 mo (%)	6 mo to 1 yr (%)	1-2 yr (%)	2-5 yr (%)	6-10 yr (%)	>10 yr (%)
	Mean/median(95% CI)/n	Mean/median (95% CI)/n	Mean/median (95% CI)/n	Mean/median (95% CI)/n	Mean/median (95% CI)/n	Mean/median (95% CI)/n
Injured side with SD	2.48/2.5	2.32/2.35	2.61/2.66	2.25/2.33	3/3	3/3
Grade 1 (5)	2	1	1	1		
Grade 2 (90)	40	19	21	10		
Grade 3 (115)	46	22	24	15	3	5
Total (210)	88 (41.9%)	42 (20%)	46 (21.9%)	26 (11.9%)	3 (1.4%)	5 (2.9%)

*SD*, scapular dyskinesis; *CI*, confidence interval.

Statistically significant *P* < .05.

## Discussion

### Prevalence of scapular dyskinesis in patients with shoulder injury

We found an SD prevalence of 97.6% (N = 210) in patients with shoulder impingement syndrome, rotator cuff pathologies, SLAP, adhesive capsulitis, and recurrent dislocation. The Grade 3 rate in the injured shoulder was 54.7%, whereas that in the noninjured shoulder was 6.7%. Shoulder impingement syndrome, rotator cuff pathology, and shoulder instability are associated with SD.^16^ Patients with degenerative rotator cuff tear exhibited an SD prevalence rate of 65.7%.^39^ At the 1-year follow-up, 100% of patients with rotator cuff repair showed SD,^20^ and 70.6% of those with AC joint dislocation exhibited SD.^9^ All patients (100%) with shoulder impingement syndrome reported winged scapulae, but SD was not investigated.^46^ Adhesive capsulitis showed altered scapular motion, but SD was not evaluated.^7^ In athletics, SD has been widely investigated and compared with shoulder injuries. Rugby players, boxers, Brazilian Ju-jitsu, and swimmers comprised 79%,^14^ 52.7%,^13^ 64.6%,^12^ and 82% of those with SD, respectively.^25^ Computer office workers and housekeepers had SD rates of 89.9%^28^ and 22.38%, respectively.^40^ SD may initiate or be associated with shoulder injuries;^16^ however, the investigation between shoulder injuries and SD is limited. Our study showed a very high SD prevalence rate in patients with shoulder injuries compared with previous studies on shoulder injuries, athletes, and other SD prevalence rates.

We identified an SD prevalence of 97.6% in injured shoulders compared with 69% in noninjured shoulders. Myers et al^29^ reported scapular malposition in baseball athletes due to chronic adaptation caused by throwing. Moreover, Myers et al^30^ reported that SD was not a risk factor for shoulder and elbow injury in high school baseball athletes. However, the investigation was performed during only one season. Plummer et al^35^ reported no significant differences in the SD prevalence between the shoulder pain and no-pain groups and concluded that SD is not more prevalent in shoulders with pain than in those without. However, the pain in the shoulder pain group was >2 out of 10 on the visual analog scale, and no specific shoulder injuries were included. Although we did not investigate the pain level in our patients with shoulder injury, our results showed a much higher SD rate in injured shoulders than in noninjured shoulders. SD might be a response to shoulder injury and cause pathomechanics to increase the rate and grade of SD.^16^ This response could be described as an adaptation from shoulder injury,^29^ as shoulder impingement, rotator cuff pathology, shoulder instability, and adhesive capsulitis are associated with SD.^24^ SD is not an injury, but it can impair healing of the shoulder injury.^31^ Therefore, SD in patients with shoulder injury could cause or be related to shoulder injuries.

### Grade of scapular dyskinesis in patients with shoulder injury

We identified a significantly higher grade of SD in injured shoulders than in noninjured shoulders. Rotator cuff tears with SD showed more significant functional impairment than rotator cuff tears without SD.^21^ The stability of glenohumeral joint needs the stable base of periscapular muscles attached on the scapula.^17^ Shoulder injuries may create pathomechanics that increase the dysfunction of periscapular muscles.^9^ Scapular kinematic alteration with rotator cuff injuries is related to loss of strength and function of the rotator cuff.^7^ Grade 3 has different scapular kinematics,^41^ and that with subacromial impingement showed reduced scapular external rotation and increased upper trapezius activity, alternating the biomechanics of shoulder movement, possibly causing the impingement.^22^ Moon and Kim^28^ identified a significantly higher neck visual analog scale score, neck disability index, and shoulder pain score with Grade 2 and Grade 3 than with Grade 1 among computer office workers. Tooth et al^42^ reported a significant decrease in accuracy and velocity of tennis serves among participants with Grade 2 and Grade 3, and they mentioned that fatigue might disturb proprioception of the periscapular muscles causing the phenomenon. They also revealed that participants with Grade 3 showed more decreased accuracy and velocity of tennis serves than those with Grade 2. Participants with Grade 3 showed decreased shoulder ROM and strength compared with those with Grade 2 SD during handball.^3^ In addition, Longo et al^21^ reported that SD patients with rotator cuff tear showed significantly decreased shoulder flexion, extension, abduction, and external rotation compared with rotator cuff tear patients without SD; thus, SD could further reduce the shoulder function of patients with rotator cuff tear. The previous studies showed decreased function with SD and that Grade 3 worsens the shoulder function. Our results support the notion that Grade 3 in injured shoulders affects the shoulder negatively compared with Grade 2 in noninjured shoulders. SD is a potential impairment in the recovery of shoulder injuries; therefore, evaluation and treatment of SD are recommended to treat shoulder injuries.^19^^,^^31^

### Chronicity of shoulder symptom and scapular dyskinesis

Although the sample size was small in the >6-year group (n = 9), the chronicity of shoulder symptom might have been related to the grade of SD in patients with shoulder injury. Koçak et al^20^ reported that the chronicity of shoulder symptoms may have decreased the strength and biomechanics of the scapular stabilizer muscles and caused SD in patients after rotator cuff repair. The periscapular muscles are affected by shoulder injuries, and dynamic stabilization of the scapula is decreased, causing problems with performing normal scapula stabilization.^16^ Madsen et al^25^ assumed that prolonged fatigue of shoulder muscles may lead to further inhibition of the scapular stabilizers, causing SD in swimmers. Baseball pitchers with SD showed more decreased shoulder function at the end of the season than at baseline.^43^ This study showed 100% Grade 3 in patients with shoulder patient at >6 years. Therefore, the chronicity of shoulder injury symptoms may worsen SD grade in patients with shoulder symptom.

### The importance of scapular dyskinesis measurement in patients with shoulder injury

This study identified a very high prevalence of SD (97.6%), with patients with Grade 3 (54.7%). Appropriate scapular alignments and movements are associated with effective shoulder function,^15^^,^^19^ and identifying the impairments of scapular kinematics can provide fundamental knowledge for the treatment of SD.^22^ SD could be a response to shoulder injury and negatively affect the shoulder injury.^19^ We identified 100% Grade 3 in patients with prolonged shoulder injury. Therefore, an appropriate diagnosis of SD is necessary to develop a treatment for such patients.^5^ This might be one of the keys to treating patients with chronic shoulder injury. Further studies are necessary to diagnose SD in patients with chronic shoulder injury and treat shoulder injuries and SD.

## Limitations

This study has some limitations. First, we identified SD in patients with shoulder injury by using the SDT; however, this test relies on the clinicians' subjective decision based on their practice experience and knowledge.^37^ Although the reliability of the SDT is almost perfect (*k* > 0.81),^36^ there is no gold standard test for SD. Second, we excluded patients who were unable to undergo a SDT because of limited shoulder ROM. Patients with severe and/or multiple shoulder injuries tend to have limited shoulder ROM. The SDT requires a shoulder flexion of 180°,^26^ which might be a barrier to testing for SD in patients with shoulder injury. The lateral scapular slide test does not require shoulder abduction >90°,^38^ but it is not suitable for diagnosing SD.^32^ Noninjured shoulders should be assumed to have Grade 1 for the lateral scapular slide test;^33^ however, we identified a SD prevalence of 69% in the noninjured shoulder among patients with shoulder injuries (Table III). Thus, further studies are required to diagnose SD patients with shoulder injury and limited ROM. Third, we excluded patients in whom both shoulders were injured owing to the study purpose. Fourth, the period of shoulder injury was obtained via the patient recall, so there might have been bias. Further study is necessary to identify the chronicity, prevalence, and grade of SD in patients with shoulder injury. Finally, the sample size was limited. Future studies are needed to identify the relationship between SD and shoulder injuries in a larger cohort.

## Conclusion

We identified a very high SD prevalence (97.6%) in patients with shoulder injury, and the prevalence of SD in the noninjured shoulders of patients with SD was lower (69%). Moreover, a much higher rate of Grade 3 was identified in injured shoulders (54.7%) than in noninjured shoulders (6.7%). Therefore, the high prevalence of SD and severe SD may be characteristic of patients with shoulder injury. Lastly, patients with chronic shoulder symptom for >6 years showed 100% Grade 3. It is recommended to diagnose and treat SD in patients with shoulder injury; however, further studies are necessary to identify the role of SD in shoulder injuries and develop effective treatment protocols.

## Disclaimer

Funding: No funding was received.

Conflicts of interest: The authors, their immediate families, and any research foundations with which they are affiliated have not received any financial payments or other benefits from any commercial entity related to the subject of this article.
